# Successful conservative treatment of a mandibular unicystic ameloblastoma: 13-year follow-up 

**DOI:** 10.4317/jced.54897

**Published:** 2018-11-01

**Authors:** Cristina-Pereira Isolan, Andressa-Goicochea Moreira, Adriana Edges, Leticia-Kirst Post, Juan-Pablo Aitken-Saavedra

**Affiliations:** 1 Graduate Program in Dentistry, School of Dentistry, Federal University of Pelotas, Pelotas, Brazil and not Post Graduate Program in Dentistry, School of Dentistry, Federal University of Pelotas, Pelotas, Brazil; 2 Department of Surgery and Maxillofacial Traumatology, School of Dentistry, Federal University of Pelotas, Pelotas Brazil; 3 Department of Oral Pathology and Medicine, School of Dentistry, University of Chile, Santiago, Chile

## Abstract

Ameloblastoma is an uncommon, locally aggressive benign odontogenic tumor and can reach considerable dimensions causing facial deformity and functional impairment. They are characterized by local aggressiveness. It is recommended that maxillary ameloblastomas be treated aggressively due to proximity of various vital structures. Conservative treatments such as marsupialization, enucleation and curettage while preserving bone integrity seem to be associated with a high rate of recurrence. Treatment evaluation of ameloblastomas is a complex issue, as ideally it should not be so destructive due to the benign nature of this lesion, but should be extensive enough to avoid recurrences. The present study is about a clinical case of a 16-year- old man with a unicystic ameloblastoma treated successfully with marsupialization. Patient was followed up every 12 months. About 13 years after diagnosis, the patient is clinically healthy and radiographically it is possible to observe evidence of bone repair.

** Key words:**Odontogenic tumors, ameloblastoma, marsupialization, unicystic.

## Introduction

Ameloblastoma is an uncommon, locally aggressive benign odontogenic tumor and can reach considerable dimensions causing facial deformity and functional impairment. Corresponds to approximately 10% of tumors affecting the mandible and maxilla. It is usually found in young adults, reaching equally men and women, with peak incidence in the third and fourth decade of life and is most often found in the body and branch of the mandible, but can occur anywhere in the mandible or jaw (1). When intraosseous, ameloblastomas can be classified as multicystic / solid or unicystic. The multicystic / solid is more common, aggressive and has a higher rate of recurrence after surgery (2).

Unicystic ameloblastoma is a variant of ameloblastoma with a relatively benign biologic behavior and better response to conservative treatment. It mostly occurs in a younger age group, the predominant location is the mandible, is radiologically unilocular, and is histologically characterized as a cystic lesion lined by an ameloblastomatous epithelial lining (2,3). Histologically, unicystic ameloblastoma is categorized into luminal, intraluminal, and mural subtypes and recurrence rate is less than 25% for all types of unicystic ameloblastomas. Although the unicystic is less aggressive than multicystic / solid and may respond well to enucleation and curettage (3), it is recommended that ameloblastomas in the jaws in general, be treated aggressively due to proximity of various vital structure (2).

In general, ameloblastomas are characterized by local aggressiveness. Conservative treatments such as marsupialization, enucleation and curettage, while preserving bone integrity and allowing continuous growth of the mandible (4) seem to be associated with a high rate of recurrence. Some articles report 90% recurrence rates for ameloblastomas that were not treated radically (5). Treatment evaluation of ameloblastomas is a complex issue, as ideally it should not be so destructive due to the benign nature of this lesion, but should be extensive enough to avoid recurrences. Several studies have reported a higher rate of recurrence after conservative treatment compared to radical treatment (6,7).

The therapeutic approach of ameloblastoma should be analyzed after a thorough analysis of different histological factors, clinical characteristics and behavior of the lesion. The most appropriate treatment is resection with a margin of 1-1.5 cm apparently unbound bone (8). Cases treated surgically can present a serious psychological trauma for those affected (4). The present study is a clinical case of a unicystic ameloblastoma treated successfully with marsupialization and whose follow-up after 13 years, is evident bone repair, without recurrences.

## Case Report

**Figure 1:**
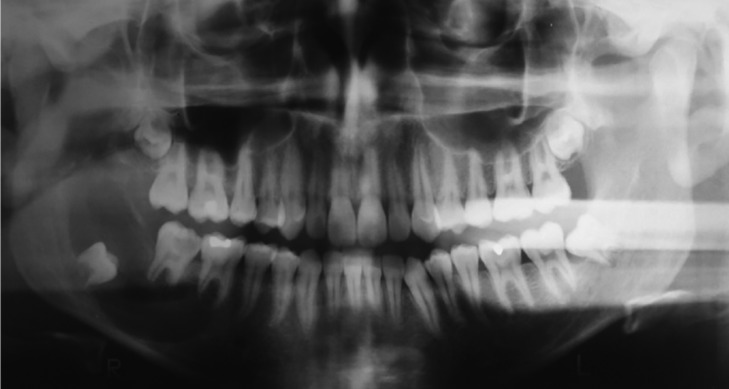
Panoramic radiography performed before marsupialization of the lesion in which the presence of the radiolucent, unilocular lesion associated to the tooth is observed 48.

A 16-year-old man with no systemic diseases, resident of the city of São Lourenço, Rio Grande do Sul, Brazil, sought a dental surgeon for the endodontic treatment of the tooth 47. Radiographs were requested in December 2004 and was observed a well delimited unilocular lesion associated with impacted tooth 48 (Fig. 1). The man was referred to a specialist in oral and maxillofacial surgery in the city of Pelotas, RS, before endodontic treatment.

**Figure 2:**
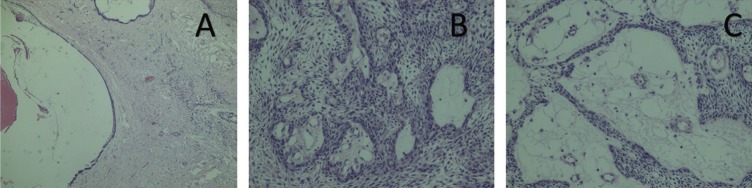
A) (4X). Histologic analysis revealed a cystic cavity lined partially coated by non-keratinized fine odontogenic epithelium with two to four layers of rounded and flattened cells, in which the basal cells were columnar, hyperchromatic, and palisaded and reversed polarity. B) (10X) and 2C (20X). Histologic analysis revealed nests and islands with ameloblastic characteristics permeated the capsular tissue of dense fibrous connective tissue.

**Figure 3:**
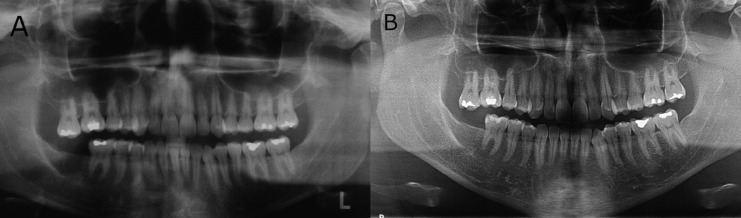
A) Panoramic radiography showing aspect of normality in the area repair process after 7-year follow-up. B) Panoramic radiography showing aspect of normality in the area repair process after 13-year follow-up.

The marsupialization of the lesion and an incisional biopsy was performed and histologic analysis revealed dentigerous cyst. Three months later the endodontic treatment of the tooth 47 was made. After nine months, the lesion was completely removed and the biopsy was sent for histopathological analysis in Center of Diagnosis of Diseases of the Mouth, Federal University of Pelotas, Brazil (CDDB-FO / UFPEL). Histologic analysis revealed unycistic mural ameloblastoma (Fig. 2A-C). Approximately 3 months after tooth 48 extraction and total removal of the lesion, it was possible to observe a mineralize d aspect compatible with the normal repair process of the region. Patient was followed up every 12 months. The tooth 47 was extracted. Currently, the patient is clinically healthy. In panoramic x-rays at 5 (Fig. 3A) and 13 years (Fig. 3B) after diagnosis it is possible to observe evidence of bone repair.

## Discussion

It is well known that the therapeutic management of ameloblastomas is a complex issue as the surgery should be invasive/aggressive to avoid subsequent recurrence. The conservative approach may consist of enucleation or curettage, sometimes preceded by marsupialization (9). The rate of recurrence after conservative treatment is a problem which has not yet been solved. Nevertheless, this study showed a successfully marsupialization treatment of an unicystic ameloblastoma with evidence of bone repair after 13 years in a sixteen years old young man.

The decision of the type of treatment of an ameloblastoma, should consider the histological pattern, clinical characteristics and behavior of the lesion, in the maxillary bones, size, age of the patient among other factors (5). However, it is established that the most appropriate treatment to prevent recurrences and possible more aggressive surgeries in the future is bone resection with a margin of 1-1.5 cm of apparently unaffected bone (8). It is important establish the histological subtype of ameloblastoma to decide an adequate treatment. It is also possible to establish erroneously, diagnosis of dentigerous cyst due to its histological similarity as it happened in this case. This diagnostic difficulty has been discussed in the literature (10) The luminal and intraluminal varieties, limited by a fibrous wall constituted by fibrous connective tissue, can be eliminated completely with a relatively good prognosis. For mural pattern (like this case) and invasive cases, it is recommended to perform surgeries with a wide margin of safety because ameloblastomas invade the trabecular spaces of bone (5,9). Some studies provide evidence that Conservative treatments for unicystic ameloblastomas like marsupialization in young patients have advantages and is effective in regressing the lesion size, ease complete removal and preserving osseous growth (11). Some studies have evaluated more conservative therapies with good results and where it is suggested to start with this modality before performing more aggressive surgeries (12). However, there is little evidence there has been a follow up for as many years as this study reports. Enucleation is not an adequate treatment for solid and multicystic ameloblastomas due to the high rate of recurrence (60-80%). In those cases, more aggressive treatments are more indicated. It happens that sometimes the subtypes of ameloblastomas cannot be identified, therefore a more radical surgery is recommended (3). In a study performed in patients with ameloblastoma, no recurrence was observed in 11 initially treated with radical resection while recurrences were observed in 22 cases initially treated with conservative therapy (13). According to Sampson *et al.* (14), when recurrence is present, extensive surgery is usually required. The case presented is in agreement with reports of ameloblastomas successfully treated conservatively in young people. Huang *et al.* (15) conducted a study in 15 patients with ameloblastoma, under 18, concluding that good results can be obtained with conservative surgery and that in case of recurrence, a second surgery can be successful. However, careful monitoring is very important. In this study case, they were 13 years of follow-up thus the chances of recurrence are very low.

Ameloblastomas remain one of the most controversial lesions in relation to the type of treatment. In order to decide, it should be considered the localization, size and histological type among other factors. Nevertheless, it is accepted that resection with safety margin is the best treatment to prevent recurrence. Although the evidence goes against, regarding treatment in ameloblastomas in general this study coincides with successful cases that evaluated conservative therapies in unycistics ameloblastomas (11), considered a less aggressive form of ameloblastomas and could be successfully treated by simple enucleation or less aggressive surgery. Furthermore, in the literature, recurrence after conservative treatment in this type of ameloblastomas is reported to be between 10 and 25%, however these reports do not specify the histological subtypes of the primary lesion. Due to this, on several occasions is choose resection as treatment, which may be unnecessary (3) In this case, it was demonstrated that conservative therapy in a young patient, not only preserved the bone structures in good condition, but also saved the patient from psychological trauma resulting from aggressive surgery.
